# Neural regulatory networks: challenges and opportunities in the treatment of psoriasis

**DOI:** 10.3389/fimmu.2026.1942535

**Published:** 2026-09-08

**Authors:** Dongjie Guo, Ruofan Xi, Tianyun Huang, Shaojie Yuan, Hanzhi Lu, Yi Wang, Jianyong Zhu, Wanjun Guo, Xin Liu, Fulun Li

**Affiliations:** 1 Department of Dermatology, Yueyang Hospital of Integrated Traditional Chinese and Western Medicine, Shanghai University of Traditional Chinese Medicine, Shanghai, China; 2 Department of Pharmacy Research, Yueyang Hospital of Integrated Traditional Chinese and Western Medicine, Shanghai University of Traditional Chinese Medicine, Shanghai, China; 3 Department of Dermatology, Shuguang Hospital Affiliated to Shanghai University of Traditional Chinese Medicine, Shanghai, China

**Keywords:** multiple cellular components, neural regulatory, neuroimmune interactions, psoriasis, therapeutic targets

## Abstract

Psoriasis is a chronic systemic inflammatory skin disease characterized by complex pathogenic mechanisms involving various cellular interactions. The nervous system significantly influences the development and exacerbation of psoriatic lesions, particularly in innervated regions. Despite the recognized importance of these interactions, a comprehensive understanding of the underlying mechanisms between different cell types and the nervous system in psoriasis is still lacking. This review systematically explores the intricate regulatory interactions between multiple cellular components and the nervous system in psoriasis. We aim to identify potential therapeutic targets and propose innovative strategies to improve clinical outcomes of patients with psoriasis.

## Introduction

Psoriasis is a prevalent chronic inflammatory dermatosis characterized by a recurrent course and complex pathogenesis (1–4). Contemporary theories primarily attribute its pathogenesis to abnormal immune activation, particularly involving T lymphocytes (especially T Helper 1 (Th1) and T Helper 17 (Th17) cells) and dendritic cells. This dysregulation results in an amplified inflammatory response, leading to excessive keratinocyte proliferation (2). Central to this process are cytokines, including interleukin (IL)-17, IL-22, tumor necrosis factor-alpha (TNF-α), and interferon-gamma (IFN-γ), which form a complex immune network that collectively drives the pathogenesis of psoriasis (2, 5, 6). Additionally, genetic predispositions, together with environmental factors such as infections, stress, and certain medications, has been demonstrated to trigger or exacerbate the condition (3, 7, 8). In clinical practice, therapeutic interventions often involve immunosuppressants like methotrexate and cyclosporine, which reduce inflammation by inhibiting T cell activity (7, 9). Furthermore, biologic agents, such as IL-17A inhibitors and TNF-α inhibitors, are used to achieve broader immune modulation and enhance therapeutic efficacy (3, 10). Despite the significant efficacy of these treatment strategies, several challenges and limitations persist. These include disease relapse following discontinuation of therapy, paradoxical eczematous reactions during treatment, and exacerbation of pre-existing lesions (11, 12). Such phenomena suggest that, in addition to the well-established immune mechanisms, other regulatory pathways may contribute to the progression of psoriasis. Therefore, identifying novel therapeutic targets to improve and optimize current treatment approaches has become an urgent priority.

In recent years, the role of nerves in dermatological health has garnered increasing attention. Skin is densely innervated by an extensive neural network (13). In healthy skin tissue, this network is widely distributed (14) and primarily responsible for essential physiological functions such as sensory conduction, pain perception, thermoregulation, and vascular tone modulation (15–18). Sensory and autonomic nerves communicate with skin and immune cells through neurotransmitters and neuropeptides, contributing to skin homeostasis (19–22). Notably, in diseases such as atopic dermatitis, sensory nerves release neurotransmitters like glutamate and neuropeptides such as substance P (SP) and calcitonin gene-related peptide (CGRP), which can exacerbate inflammation and itching (23–25). Further investigation into neural regulatory mechanisms in skin inflammation may provide novel insights for treatment.

An increasing number of studies have highlighted the association between the nervous system and psoriasis, revealing several important coexisting phenotypes that suggest a bidirectional relationship (1, 26). On one hand, nerves may influence the onset and progression. Psoriatic lesions exhibit dense nerve endings and neural infiltration (27–31), and neuropeptides such as SP and CGRP released from sensory nerve terminals promote inflammatory cell recruitment and cytokine production (32, 33), exacerbating the condition. Additionally, an increased sympathetic nervous activity and hyperactivation of the hypothalamic-pituitary-adrenal (HPA) axis may further aggravate psoriasis (34). Notably, nerve damage has been reported to improve or even resolve psoriatic lesions (35). On the other hand, psoriasis may also affect the nervous system. Key inflammatory molecules such as IL-17 and IL-23 are involved in sensory nerve signaling and can influence synaptic plasticity and blood-brain barrier integrity. IL-17 may promote neuronal growth and repair, while IL-23 may modulate nerve fiber density and functionality (1). These findings underscore a closer connection between the nervous system and psoriasis than previously recognized. Studying nerve-cell interactions in psoriasis is crucial for uncovering new pathogenic mechanisms and may offer insights for novel therapeutic strategies. However, research in this area remains in early stages, and the regulatory network within the psoriatic microenvironment is not yet fully understood.

**Figure 1:**
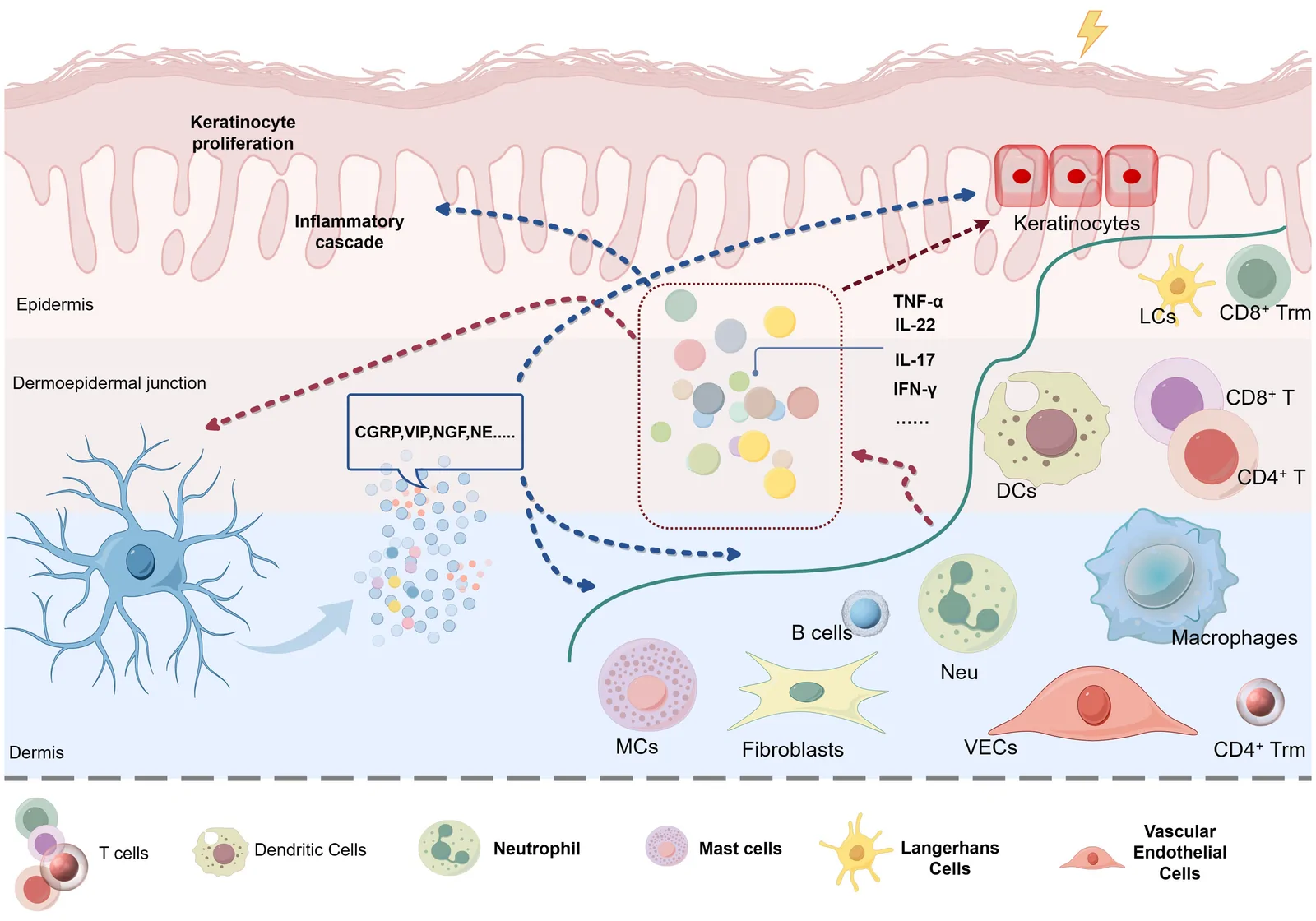
Psoriasis skin microenvironment. A schematic overview of the neuro-immune regulatory network in psoriatic skin is shown in the image. Sensory, sympathetic, and autonomic nerve fibers interact with immune cells through neurotransmitters and neuropeptides including CGRP, VIP, NGF and NE etc. These neural signals create a bidirectional communication network that modulates inflammatory cascades and keratinocyte responses in psoriasis through cytokines, namely TNF-α, IFN-γ, IL-22, and IL-17. CGRP, calcitonin gene-related peptide; VIP, vasoactive intestinal peptide; NGF, nerve growth factor; NE, norepinephrine. Created with Figdraw.

This review aims to comprehensively outline the complex relationships between various cell types and the nervous system in psoriasis (**Figure 1**). By examining current knowledge of nerve-cell interactions in psoriatic skin, we seek to clarify mechanisms underlying disease progression, uncover underexamined aspects of pathogenesis, provide a theoretical foundation for further investigation, and inform the development of innovative treatment strategies. We will address the following key aspects: first, interactions between nerves and immune cells in inflammation regulation; second, connections between the nervous system and keratinocytes, fibroblasts, and other cell types; and finally, potential therapeutic approaches based on specific mechanisms of cellular interaction.

## Methods

### Search strategy and selection criteria

The search was conducted in the PubMed database using MeSH terms. The search terms included (nervous system OR neural network OR neuroimmunity OR neuropeptide OR neurotransmitter OR sensory neuron OR sympathetic nerve OR central nervous system OR parasympathetic nerve OR Neuro-) AND (psoriasis) AND(immune cells OR keratinocytes OR T cells OR dendritic cells OR macrophages OR fibroblasts OR vascular endothelial cells OR Langerhans cells OR mast cells OR cytokines OR interleukins OR extracellular vesicles OR exosomes OR traditional Chinese medicine OR Chinese herbal medicine OR herbal medicine OR phytotherapy), with a primary focus on literature published within the past 5 years. All retrieved articles were screened in a double-blind manner by two reviewers, retaining primarily reviews, clinical trials, and basic research studies, with a third reviewer conducting a final review. Data analysis was performed on eligible studies, focusing on key findings related to the neuromodulation of psoriasis.

### Interactions between different cells and nerves

The primary cell types commonly involved in psoriasis include various immune cells, keratinocytes, fibroblasts and other effector cells. Together with neuronal cells, these cells form a neurocutaneous network mediated by neurotransmitters, neuropeptides, cytokines, regulatory RNAs, and extracellular vesicles (EVs). These signaling mechanisms are discussed below in a cell-specific context.

### Immune cells

Immune cell mechanisms are crucial in psoriasis pathogenesis. While traditional models focus on cytokine networks (8, 36), immune-neural cell interactions also drive its progression, affecting inflammation, lesions, and neural functions.

#### T cells

**Figure 2:**
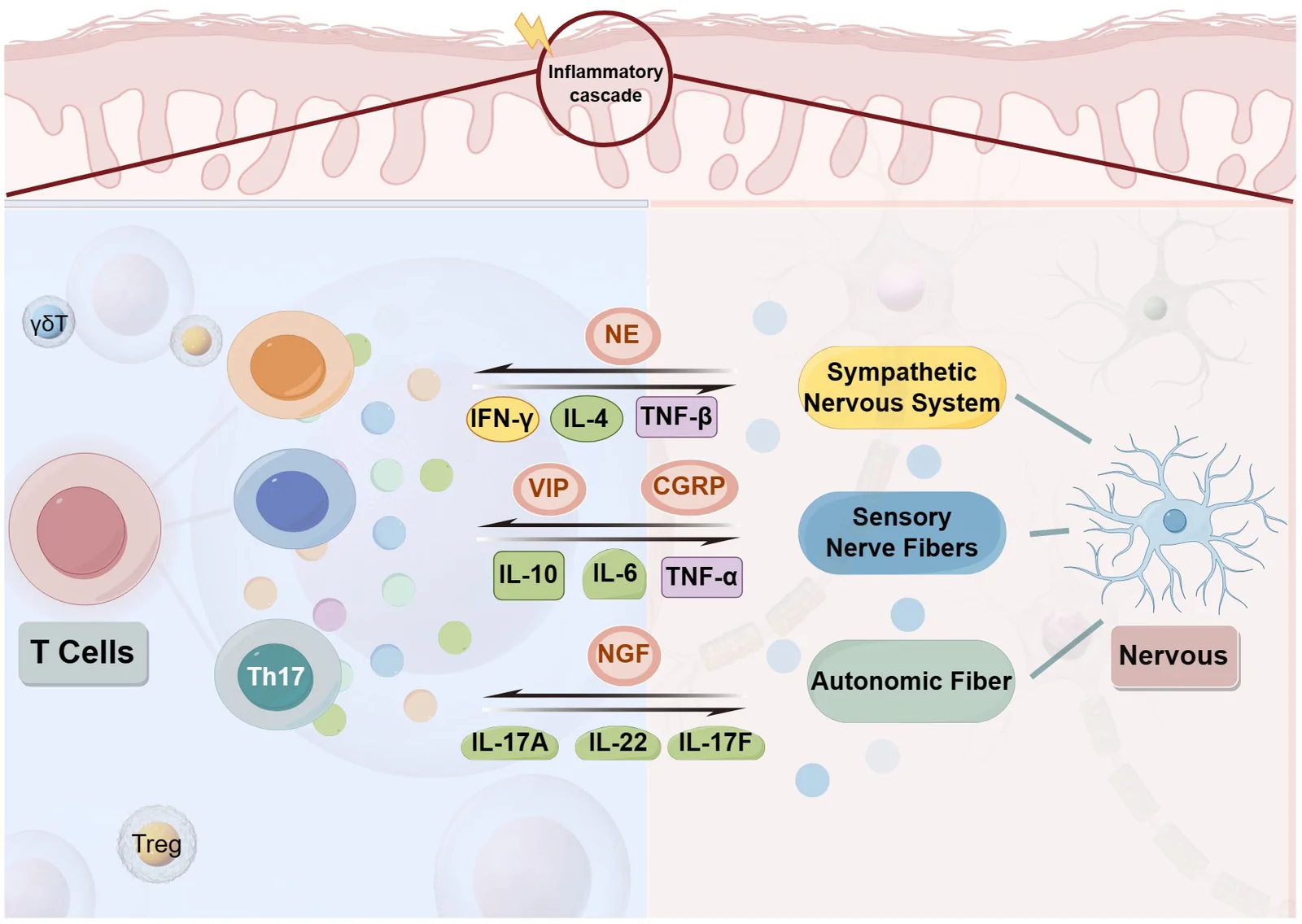
T cell interactions with the nervous system. Nerve fibers release neurotransmitters and neuropeptides to regulate T-cell differentiation and activation. These neural signals differentially modulate T-cell responses according to the nerve type and neural mediator involved. T-cell-derived cytokines and mediators can in turn affect neuronal activity and sensitivity, forming bidirectional neuroimmune communication. Created with Figdraw.

T cells play a central role in the pathogenesis of psoriasis (37). Recent research has further revealed that T cell-neural interactions play a crucial role in the progression and recurrence of psoriasis, offering new insights for a more comprehensive understanding and response to the disease (**Figure 2**).

Distinct neural pathways differentially regulate T-cell activation and differentiation. Studies on sympathetic nerves have shown that their distribution interferes with T cell homeostasis (38). Norepinephrine (NE), released from sympathetic nerves, binds to Th1 cells, thereby regulating the levels of cytokines such as IFN-γ and TNF-β (39). Cutaneous calcium/calmodulin-dependent protein kinase II-γ (CAMK2γ), primarily expressed in sympathetic nerves, exacerbates psoriasis symptoms through activation of the NE-γδ T cell-adrenergic receptor β1-nuclear factor-κB and -p38 axes, acting on γδ T cells and promoting the production of IL-17 (40).

Neuropeptides released from sensory and autonomic nerve fibers can also modulate T-cell differentiation. Vasoactive intestinal peptide (VIP), which is present in sensory and autonomic nerve fibers in the skin (41, 42), reduces pathogenic Th17, Th1, and Th17/1 profiles (43). CGRP also plays a key regulatory role in skin immune and inflammatory processes (44, 45) and coordinates T-cell differentiation (46). For example, the interaction of receptor activity-modifying proteins 3 (RAMP3) expressed on T cells with CGRP promotes the production of Th1 and CD8^+^ T cells (46). CGRP specifically binds to the corresponding receptor on the surface of Th2 cells, subsequently activating intracellular signaling pathways such as Phosphoinositide 3-kinase/Protein Kinase-B(PI3K/Akt), which regulate the transcriptional levels of relevant genes in Th2 cells and ultimately influence the secretion of IL-4, IL-10, and other cytokines (47, 48). CGRP signaling, following denervation, prevents the mediated reduction of CD4^+^ T cells (48), biasing the immune response toward Th17 polarization, which, in turn, affects antigen presentation to T cells by Langerhans cells (49). Nerve growth factor (NGF) directly regulates both intrinsic and adaptive immune responses in T cells through p75 neurotrophin receptor(p75NTR) and the tropomyosin-related tyrosine kinase(Trk) receptors, leading to the release of neuropeptides and neurotransmitters that control immune system activation in inflammatory tissues (50). In psoriasis, NGF attracts T cells to infiltrate lesion sites, creating a cascade of amplified inflammatory responses (51).

Conversely, T cells can influence neural function and phenotype. Activated T cells release pro-inflammatory cytokines, such as TNF-α, which can directly affect neurons (52). This cytokine-mediated regulation also involves IL-17A and oncostatin M (OSM). In psoriasiform models, IL-17A acts directly on IL-17RA-expressing sensory neurons, inducing neuronal IL-6 and downstream astrocytic IL-1β release and promoting maladaptive cutaneous hyperinnervation (53, 54). OSM, which is increased in psoriatic skin and produced mainly by dermal T cells, sensitizes OSMR-expressing sensory neurons and enhances neuronal excitability (55). Memory Th2 cells synergize with sensory neurons that express CGRP. Th1 cells interact with neurons to produce differentiation- promoting CGRP via the RAMP3/calcitonin receptor-like receptor (CALCRL), whereas the opposite occurs for Th2 cells (45).

This bidirectional interaction highlights the interdependence between T cells and nerves in the pathological processes of psoriasis, forming a persistent inflammation-neural feedback loop, which may be a crucial mechanism for disease persistence and recurrence.

#### Dendritic cells

Dendritic cells are key antigen-presenting cells that initiate the immune response in psoriasis and sustain disease progression (56). Previous studies have demonstrated that dendritic cells efficiently activate T cells and contribute to the immune response through their antigen-presenting capacity (57). In skin tissue, sympathetic nerve fibers are in close proximity to dendritic cells (58), and an increasing body of research suggests that dendritic cells also interact with neuromodulatory networks.

Nerves can influence the signaling and immunomodulatory functions of DCs by releasing chemical signals. For example, when sympathetic nerves are activated, the released NE acts on the β2-adrenergic receptor (β2-AR) on the DCs surface to increase intracellular cyclic adenosine monophosphate (cAMP) levels via stimulatory subtype of G protein (Gs) protein coupling (59), which further activates protein kinase A (PKA), inhibits nuclear factor-κB(NF-κB) and activator protein 1(AP-1) in the DCs, and modulates effector T cell differentiation (60). Vasoactive intestinal peptide (VIP), released by autonomic and sensory nerve fibers (61), regulates broad-spectrum inflammatory mediators both directly through activated macrophages and via the production of interleukin 10-secreting regulatory T cells, which have inhibitory capacity for self- reactive T cells (62). In addition, nerves can also influence the local inflammatory response and adaptive immune response of DCs through physical contact and electrical activity, such as by modulating DCs via the release of CGRP, triggering contact-dependent Ca2^+^ fluxes and membrane depolarization, and by secreting the chemokine monocyte chemoattractant protein-2 (CCL2), which leads to neurogenic inflammation in barrier tissues (63–68). More specifically, nociceptive neurons, including transient receptor potential vanilloid 1(TRPV1)^+^Nav1.8^+^ fibers, drive IL-23 production by dermal DCs, with CGRP acting as an important mediator of this pathway and promoting downstream IL-17A/IL-22 responses from γδT cells (66). Beyond classical neuropeptides, activity-dependent release of miR-let-7b from dorsal root ganglion neurons can activate Toll-like receptor 7 (TLR7) on DCs and enhance the IL-23/IL-17 inflammatory axis (33, 69).

DCs also modulate neurological function in psoriasis. It has been found that DCs increase the excitability of sensory nerves by increasing the production of IL-23 through the cAMP-dependent pathway under the action of prostaglandin E2 (PGE2), thereby making patients more sensitive to pain sensation (70, 71). In addition, DCs attract inflammatory cells to gather around the nerves under the action of CCL2, which further affects the microenvironment of sensory nerves, leading to nerve damage or abnormal function (58). Dendritic cells respond to nerve signals in psoriasis while counter-regulating nerve function, forming a complex network of neuroimmune interactions.

#### Macrophages

M1-type macrophages are elevated in the peripheral blood of patients with psoriasis (72), and studies in mice have shown that macrophage depletion is generally believed to ameliorate psoriasis inflammation, and reduce levels of Th1 cytokines (including IL- 1a, IL-6, IL-23, and TNF-α) to normal levels (56, 73–76).

In recent years, with the advancement of research, the regulatory role of the nervous system in macrophages has gradually been elucidated. Sensory nerves can modulate macrophage inflammatory and repair-associated functions through neuropeptides (77). For example, CGRP acts on various cells through receptor activity modifier protein 1 (RAMP1), inhibiting macrophage recruitment, accelerating cell death, enhancing efferocytosis, and promoting polarization toward a pro-repair phenotype (78). In psoriasis, IL-23 can induce a distinct M(IL-23) macrophage state that promotes psoriasiform inflammation (79, 80). The neuropeptide TAFA chemokine like family member 4(TAFA4), produced in the skin by C-low threshold mechanoreceptors, promotes IL-10 production by dermal macrophages, ensuring their survival and maintenance, reducing skin inflammation, and promoting tissue regeneration (77). Furthermore, somatostatin (SST), released from sensory nerve endings, can promote the production of the immunosuppressive cytokine IL-10 by binding to the somatostatin receptor 5(SSTR5) receptor expressed on macrophages (81). Beyond neuropeptides, sensory neurons can also regulate macrophages through non-coding RNAs. Stress-associated activity-dependent release of miR-let-7b from sensory neurons activates TLR7 on skin-resident macrophages, increasing inflammatory mediator production and enhancing the IL-23/IL-17 axis (82).

Conversely, macrophages can regulate sensory neuronal output through cytokines. In imiquimod (IMQ)-induced psoriasiform mice, IL-1β released by M1-polarized dorsal root ganglion macrophages induces mitogen-activated protein kinase (MAPK)-dependent IL-6 expression in sensory neurons, followed by neuronal IL-6 release into the skin, thereby exacerbating cutaneous inflammation. Thus, bidirectional macrophage–nerve interactions may contribute to inflammatory persistence or resolution, depending on the macrophage state and local microenvironment (83).

### Other immune cells

Although the roles of mast cells, B cells and neutrophils in psoriasis have received relatively less attention, recent studies suggest that they may have unique functions in neuro-immune interactions, which potentially play essential roles in psoriasis exacerbation or recurrence.

Mast cells (MCs) play a crucial role in the onset, progression, and maintenance of psoriasis through interactions with T cells, Tregs, keratinocytes, adipocytes, and sensory neurons (84). As previously mentioned, sensory neurons secrete various neuropeptides that contribute to the immune response in psoriasis, and MCs can be activated by neuropeptides (85, 86). MC-neuron interactions also occur in psoriasis. In psoriatic lesions, levels of SP, nerve density, the total number of MCs, degranulation, and the contact between MCs and sensory nerves are elevated (87). MCs have been shown to enhance TRPV1 sensitivity or lower its activation threshold through the release of injurious and excitatory mediators (e.g., histamine, prostaglandins, and TLRs) and initiate reciprocal communication with specific injury receptors on sensory nerve fibers (88). Corticotropin-releasing hormone (CRH), adrenocorticotropic hormone (ACTH), and glucocorticoids released by the cerebro-cutaneous axis, which regulate psoriasis, are involved in the local immune response (89), with elevated CRH inducing MC degranulation. In contrast, MC secretion of IL-6 can also stimulate CRH secretion by activating the hypothalamic-pituitary-adrenal (HPA) axis through a feedback mechanism (90). Additionally, mast cells sense tissue injury and stress signals through receptors such as MrgprB2, which contribute to the formation of sensations such as nociception (84).

Neutrophil chemotaxis, activation, and phagocytosis can all be negatively regulated in an NE-dependent manner (91). Neutrophils are a diffuse source of acetylcholine and catecholamines, and these neurotransmitters also feedback on neutrophil function (92, 93). Neutrophils contribute to the activation and sensitization of sensory neurons, particularly during injury perception and pain events. Moreover, activation of injury receptors promotes axonal reflexes, triggering local release of neuromediators and causing neutrophil activation (94–96). Treatment with NE in sympathetic nerves impairs neutrophil chemotaxis and induces an N2-type neutrophil phenotype that reduces the expression of genes critical for cytoskeletal remodeling and inflammation. Prolonged absence of NE administration promotes neutrophil release of myeloperoxidase and IL- 6 but inhibits interferon-gamma and IL-10 production and reduces neutrophil activation and phagocytosis (91). EV-mediated signaling provides an additional route of immune-cell communication in psoriasis. Neutrophil-derived EVs (97) can activate keratinocytes in generalized pustular psoriasis, whereas mast cell-derived (98, 99) EVs can activate CD1a-reactive T cells and promote IL-17A and IL-22 production. However, direct EV-mediated communication between neurons and skin cells in psoriasis remains to be established.

B cells may be triggered by autoantigens in psoriasis, inducing molecular mimicry to alter B cell activity within the germinal centers (GCs), producing autoantibodies and proinflammatory cytokines, forming ectopic GCs, and dysregulating the proliferation of keratinocytes. This suggests that B cells may induce a local inflammatory response in the development of psoriasis through the production of pro-inflammatory antibodies and may interact with nerve endings to enhance their inflammatory responsiveness (36). B-cell-derived GABA(γ-aminobutyrate) promotes the differentiation of monocytes into anti-inflammatory macrophages, secretes interleukin 10, and inhibits the killing function of CD8^+^ T cells (100).

Therefore, mast cells, neutrophils, and B cells may influence the progression and recurrence of psoriasis through complex cell-neuron interactions within the neuro- immune network. This perspective provides new insights into the study of neuro- immune regulation in psoriasis.

Currently, the specific mechanisms underlying the interactions between immune cells and nerve cells in psoriasis remain incompletely elucidated. However, insights from other diseases, such as multiple sclerosis, where the immune system attacks the myelin of the central nervous system leading to neurological impairments, provide valuable perspectives for understanding similar processes in psoriasis (101).

#### Keratinocytes

Keratinocytes, the predominant cell type in the epidermis, play a crucial role in maintaining both skin barrier and immune functions. Previous studies have shown that during the early pathogenic events and chronic inflammation associated with psoriasis, keratinocytes undergo excessive proliferation and abnormal differentiation due to immune activation (102, 103). For example, keratinocytes are involved in the recruitment and activation of plasmacytoid dendritic cells (pDCs) and neutrophils (104, 105), releasing the human cathelicidin(LL-37), which activates macrophages through recognition of the P2X7 purinergic receptor, thereby promoting their differentiation into a pro- inflammatory phenotype (106). Furthermore, LL-37 induces the secretion of Interleukin- 8(CXCL-8) and CXCL-1 chemokines via IL-36R signaling, leading to the release of IFN-α and the initiation of a monocytic Th1 response to activate T cells (107). Keratinocyte-derived EVs can also modulate immune-cell responses in psoriasis. These EVs can transfer regulatory RNAs to CD4^+^T cells and promote Th1/Th17 differentiation (108), while leucine-rich alpha-2-glycoprotein 1-enriched keratinocyte-derived EVs can promote macrophage activation in psoriasiform dermatitis (109). In recent years, the intricate interactions between the nervous system and keratinocytes have garnered increasing attention. Not only does the nervous system exert direct and indirect regulatory effects on keratinocyte function, but keratinocytes also influence the nervous system, forming an interdependent “neuro-immune-skin” regulatory network (110, 111).

In psoriasis, an increased density of nerve endings is observed in the epidermis of lesional skin, with a concentrated distribution around keratinocytes. These nerve endings release neuropeptides that bind to receptors on the surface of keratinocytes. VIP induces keratinocyte production of IL-6, IL-8, RANTES (regulated upon activation, normal T-cell expressed and secreted; CCL5), stem cell factor, and vascular endothelial growth factor (VEGF) (42, 112). In spinal cord hemisection mice, sensory deficits were found to ameliorate psoriatic dermatitis and inhibit the abnormal proliferation of keratinocytes (29). Neuropeptides such as SP and CGRP predominantly affect keratinocyte proliferation and inflammatory responses (113, 114). Furthermore, pro-inflammatory factors such as IL-6, secreted upon the binding of SP to its receptor, exacerbate local inflammatory responses (107). The nervous system also indirectly influences keratinocytes by affecting the local immune environment. Sympathetic nerves may enhance the activity of dendritic cells through the release of neurotransmitters such as NE, promoting the secretion of IL-23, which activates T cells and stimulates pro-inflammatory responses and barrier function in keratinocytes (110, 115, 116). Additionally, research indicates that the activation of sensory neurons can trigger Th17-type immune responses, further exacerbating keratinocyte proliferation and the pathological process of psoriasis (117). The role of nerves in keratinocytes in other diseases is also instructive. In wound healing, the phosphorylation of the NGF receptor TrkA inhibits keratinocyte proliferation (118), whereas stimulation through the TrkA-PI3K/Akt pathway promotes keratinocyte proliferation and neuronal outgrowth (119). This indicates that keratinocytes can influence the nervous system via factors such as NGF, suggests that NGF enhances the inflammatory signal transduction in keratinocytes, and a similar mechanism may also be applicable in psoriasis (120, 121). Furthermore, in neurofibromatosis (NF), abnormalities in nerve proliferation and function directly lead to skin lesions (122, 123). Although NF and psoriasis may not share identical pathological mechanisms, both involve abnormal interactions between nerves and skin cells, offering new perspectives for understanding these interactions in psoriasis.

Keratinocytes are capable of secreting neurotrophic factors like NGF which directly promote the growth and sensitivity of nerve endings. For instance, NGF facilitates the responsiveness of nerve endings to inflammatory signals, thereby sustaining and enhancing the sensitivity of the nervous system during the pathological process of psoriasis (124). Moreover, the expression of SP receptors and NE receptors on keratinocytes further amplifies the responsiveness of nerve endings to inflammatory signals, contributing to a more persistent local inflammatory response (125). These receptors have also been highlighted in the context of the neuro-immune mechanisms underlying psoriasis (125, 126). Additionally, chemokines and pro-inflammatory factors secreted by keratinocytes can indirectly influence neurons. For example, through IL- 36R signaling, keratinocytes induce the secretion of chemokines like CXCL-8 and CXCL-1, attracting neutrophils and macrophages to the site of inflammation and exacerbating the local inflammatory environment, which in turn enhances neuronal excitability (106, 107). In atopic dermatitis (AD), thymic stromal lymphopoietin (TSLP) secreted by keratinocytes can activate neurons, promoting not only immune cell activation and inflammatory responses but also increasing neuronal sensitivity to external stimuli, thereby intensifying pruritus and skin inflammation (127–129). This suggests that the nervous system may regulate keratinocytes in psoriasis through analogous mechanisms, further aggravating inflammatory responses.

In contrast to the bidirectional interactions observed in immune cells, the interactions between keratinocytes and nerves focus on the local cell-neuron network in the skin, emphasizing the enhanced sensitivity of nerve endings and the ability to spread local inflammation, which helps to maintain and enhance the chronicity of psoriasis. The mechanism of this bidirectional regulation is being progressively elucidated in psoriasis and validated in other diseases, and such interactions also provides new perspectives for exploring therapeutic strategies.

#### Fibroblasts

**Figure 3:**
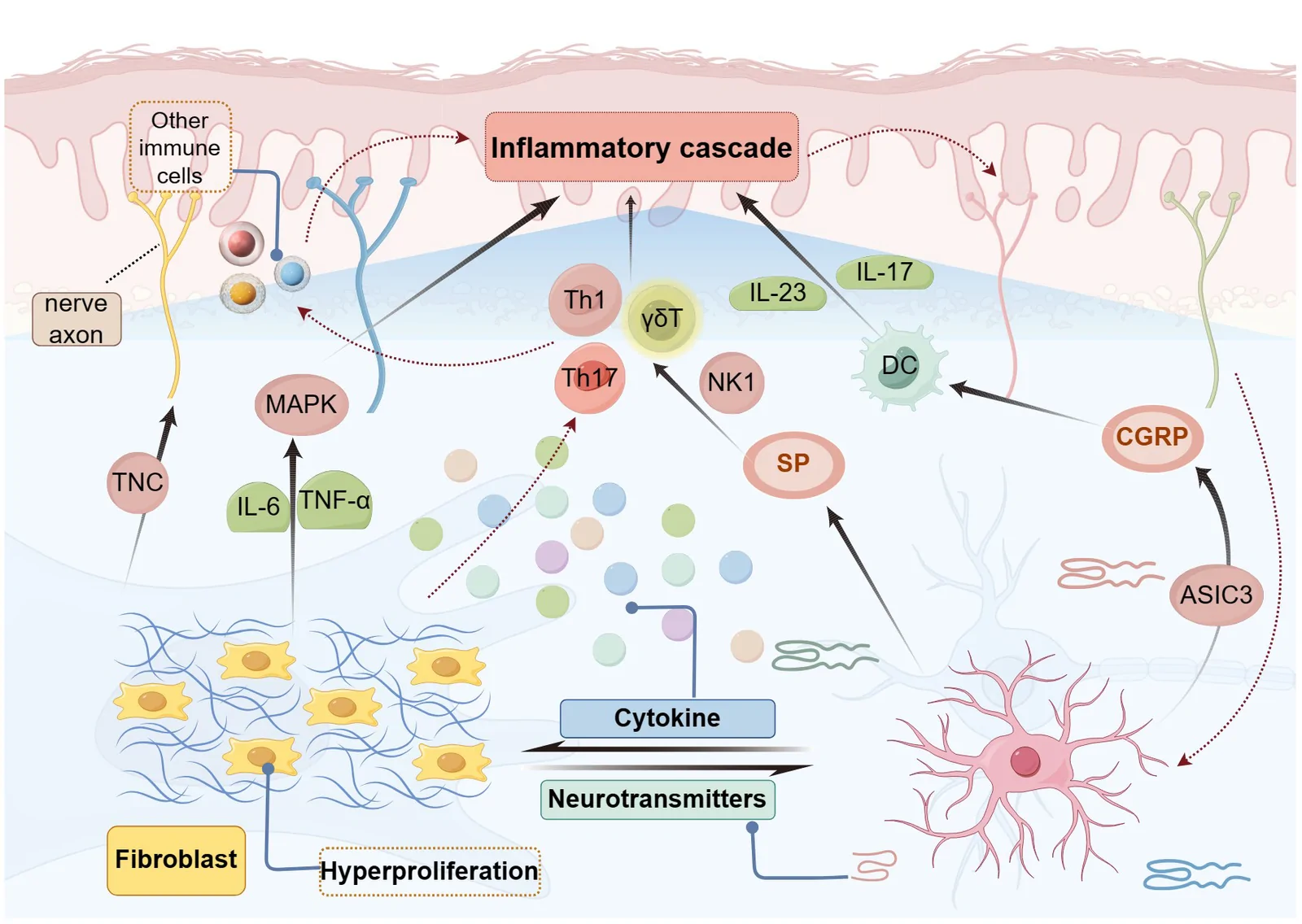
Interaction of fibroblasts with the nervous system. Fibroblasts exhibit hyperproliferation and engage in bidirectional communication with nerve cells via neurotransmitters and cytokines. Fibroblast-derived ILs activate MAPK signaling and amplify inflammatory cascades, while TNC promotes nerve axon growth. IL, interleukin; TNC, Tenascin-C. Created with Figdraw.

Fibroblasts are the primary cell type in the dermis and are well-known for their roles in extracellular matrix production and tissue repair. Previous studies have primarily focused on their involvement in tissue repair and fibrosis. Fibroblasts contribute to wound healing and scar formation by secreting collagen and various growth factors, such as platelet-derived growth factor (PDGF) and transforming growth factor-beta (TGF-β) (130–132). Recent research has revealed that fibroblasts are not only involved in tissue repair processes but also play a significant role in psoriasis. The proteomic profile of dermal fibroblasts in psoriasis shows substantial alterations, including the upregulation of pro-inflammatory and antioxidant proteins, signaling molecules, and proteases (133). These changes are thought to contribute to the excessive proliferation of keratinocytes and the recruitment of immune cells, both of which are hallmark features of psoriatic plaques (132). These findings suggest that fibroblasts play a critical role in psoriasis progression, and their interactions with the nervous system are summarized in **Figure 3**.

In the nervous system, peripheral nerves are primarily composed of axons of neurons, Schwann cells that encase these axons, and fibroblasts. Fibroblasts play a crucial role in the composition of the endoneurium, perineurium, and epineurium (134). The nervous system directly influences fibroblast behavior through the secretion of neurotrophic factors. In the inflammatory environment of psoriasis, dynamic interactions among various cytokines can elevate the expression of nerve growth factor (NGF) in fibroblasts (119). NGF induces the proliferation of local nerve fibers, resulting in an increased density of nerve endings within psoriatic lesions. This process is mediated through the NGF-TrkA signaling pathway, which directly drives the excessive proliferation of keratinocytes, a hallmark of psoriatic skin lesions (26). Studies suggest that inhibiting NGF signaling not only reduces fibroblast activation but also suppresses nerve growth, ultimately helping to alleviate inflammation and reduce the severity of symptoms (135). Inflammation also drives the emergence of a subpopulation of Tnc^+^ fibroblasts with distinct neuromodulatory properties. These cells are localized in the papillary dermis, adjacent to the epidermis, an area where fibroblasts, peripheral nerves, and T cells engage in close interactions. Tenascin-C (TNC) overexpression by fibroblasts notably enhances neurite outgrowth, a process mediated by the extracellular signal‐regulated kinase(ERK) pathway, though it does not require direct contact with the neuronal cisterna (136). SP, CGRP, and alpha-melanocyte stimulating hormone (α-MSH) influence the IL-8/IL-8 receptor system in human keratinocyte cell lines and dermal fibroblasts, indicating that neuromodulation may play a role in the chronic progression of psoriasis through a complex, multilayered regulatory mechanism (137).

In psoriasis, the regulation of nerves by fibroblasts is likely closely associated with the disease’s progression and recurrence. Fibroblasts secrete a variety of neurotrophic factors, such as brain-derived neurotrophic factor (BDNF) and fibroblast growth factor 21 (FGF21), which promote neuronal growth, differentiation, and repair, ultimately increasing local nerve sensitivity (138, 139). In psoriatic lesions, fibroblast-derived Tenascin-C (TNC) has been shown to drive the excessive proliferation of nerve endings, thereby exacerbating itching and pain symptoms in patients (136). Regarding immune response modulation, fibroblasts serve as a primary source of interleukin-6 (IL-6), a cytokine that plays a critical role in neuronal development, differentiation, regeneration, and degeneration (140). By releasing inflammatory factors, fibroblasts regulate neuronal function and development, thereby intensifying nerve-related pain and exacerbating local inflammatory responses (141–144).

The impact of fibroblasts on the nervous system has garnered attention in various other diseases. For instance, compared to cardiac-derived fibroblasts, neural-derived fibroblasts significantly promote axon growth in motor neurons *in vitro*. Brain-derived neurotrophic factor (BDNF) secreted by neural-derived fibroblasts enhances the expression of β-actin and F-actin via ERK and AKT signaling pathways, thereby promoting axon growth in motoneurons (134). Fibroblasts also play a role in neuronal transformation. In a study, activation of the PTB-REST-miR-124 loop in adult fibroblasts after knockdown of Polypyrimidine tract-binding protein(PTB) resulted in the production of only immature neurons. However, further inactivation of nPTB, an analog of PTB, led to the generation of mature neurons. Mechanistic studies revealed that inactivation of nPTB activates a distinct nPTB-BRN2-miR-9 loop, which involves nPTB, the transcription factor Brn2, and miR-9, thereby regulating neuronal maturation (145). In fibrotic conditions such as systemic sclerosis and idiopathic pulmonary fibrosis, fibroblasts become activated and adopt a myofibroblast phenotype. This phenotype is characterized by excessive deposition of extracellular matrix (ECM), contributing to tissue remodeling and fibrosis (146, 147). This fibrotic environment is often accompanied by an elevated expression of nerve growth factor (NGF) and other neurotrophic factors, which in turn promote the proliferation and activation of fibroblasts (148). Notably, NGF is upregulated in fibrotic tissues, where it promotes fibroblast survival and extracellular matrix (ECM) production through its receptor, TrkA (149). This interaction creates a positive feedback loop, where NGF not only stimulates fibroblast activity but also induces nerve growth into fibrotic tissues, further sustaining the fibrotic process and leading to chronic pain. Although the pathological mechanisms of psoriasis differ from those of tumors, these mechanisms suggest that the “neuro-friendly” behavior of fibroblasts may occur similarly in other chronic pathological environments. Fibroblasts—often referred to as cancer-associated fibroblasts (CAFs)—interact with nerves and are linked to tumor growth, metastasis, and increased pain (150), influencing tumor progression and metastasis (151, 152). Neuropathic pain conditions also involve significant interactions between fibroblasts and neurons (153). In these conditions, fibroblasts are activated and contribute to the neuroinflammatory environment, enhancing pain sensitivity. Through the release of neurotrophic factors and cytokines, fibroblasts influence neuronal behavior, including axon growth and synaptic plasticity, further exacerbating pain perception. These interactions create a cycle of ongoing pain and inflammation, with fibroblasts playing a central role in maintaining the neuropathic pain state.

The complex network between fibroblasts and the nervous system leads to recurrent or exacerbated inflammatory and pain responses in psoriasis patients as the disease progresses. This bidirectional interaction, wherein fibroblasts modulate neuronal activity and vice versa, may play a crucial role in sustaining chronic inflammation and pain. Understanding this fibroblast-neuron axis could pave the way for future interventions targeting this pathway, potentially reducing the recurrence and severity of psoriasis symptoms, and offering new therapeutic strategies for managing the disease.

## Other cells

### Vascular endothelial cells

In psoriasis, abnormalities in angiogenesis are commonly observed in the dermal layer, with vascular dilation exacerbating lesions. VECs, as the predominant cell type in the vascular inner wall, are activated and proliferate significantly in response to pro- angiogenic factors such as VEGF, TGF-β, and IL-17. VECs quickly adapt to changes in the tissue microenvironment by receiving and integrating various signals, including hormones, neurotransmitters, and shear stress (154–157).

The communication between the nervous system and vascular endothelial cells is mediated through a complex network of signaling molecules. For instance, the NGF involved in immune responses can influence VEGF expression through the PI3K/mTOR(phosphoinositide 3-kinase/mammalian target of rapamycin) signaling pathway, promoting endothelial cell proliferation and angiogenesis (158, 159). This mechanism may exacerbate inflammation in psoriatic lesions by enhancing vascular permeability and the formation of new blood vessels. Increased concentrations of neurotransmitters such as SP in affected areas stimulate the activity of vascular endothelial cells, leading to elevated expression of E-selectin and intensifying the inflammatory response of blood vessels (87). This direct regulation not only affects vascular dilation but may also further amplify local immune responses, resulting in sustained inflammation in the affected areas and contributing to the chronic progression of psoriasis.

Vascular endothelial cells directly or indirectly affect the state of nerves through the local microenvironment. In psoriasis lesions, vascular endothelial cells attract inflammatory cells and regulate the release of neurotransmitters by secreting chemokines such as IL-8, growth-regulated protein alpha(GRO-α), C5a dearginine, and inflammatory mediators, which in turn increase the density and activity of sensory nerve endings, causing symptoms such as itching and pain in affected patients (160–163). This alteration is closely associated with the exacerbation of psoriatic symptoms, as increased nerve density and heightened sensitivity are typically accompanied by more intense clinical symptoms. VECs secrete signaling molecules that promote the repair and regeneration of the nervous system, and vascular endothelial cadherin is closely related to the stability of the surrounding nerves (164). This action may play a crucial role in the recurrence and symptomatology of psoriasis. In psoriatic lesions, endothelial cells may maintain a state of nerve hypersensitivity through the aforementioned factors, leading to persistent symptoms of itching and pain in affected areas, thereby driving the recurrence of the disease.

In other conditions, synovial fibrosis activates vascular endothelial cells, promoting Netrin-1-induced sprouting of sensory nerves and increasing pain sensitivity (165). Sensory neurons, through SP signaling, directly stimulate the proliferation of vascular endothelial cells in response to inflammation (166). These findings provide insights into the indirect regulation of vascular endothelial cells by nerves in psoriasis, suggesting that similar mechanisms may be present in this condition and offering new perspectives and potential therapeutic targets for neurovascular interactions in psoriasis.

A complex feedback loop is also formed between the nervous system and the vascular endothelium, which may also point to potential therapies to target this.

### Langerhans cells

Pathological studies of psoriasis have indicated that LCs, a dendritic cell subset located exclusively within the epidermis, play a crucial role in the maintenance and progression of inflammation in psoriasis by presenting self-antigens to T cells and producing pro- inflammatory cytokines such as interferon-gamma (IFN-γ) and interleukin-17 (IL-17). This process activates Th1 and Th17 cells, leading to the hyperproliferation of keratinocytes and sustaining the chronic inflammatory environment characteristic of psoriatic lesions (167–169).

The nervous system can regulate LCs from multiple angles, firstly, LCs in the skin are adjacent to sensory nerve fibers and express neurotransmitter receptors, enabling direct communication with the peripheral nervous system (58, 169). Neuropeptides released from nerve endings bind to receptors on Langerhans cells, thereby inhibiting their antigen presentation capabilities in lymph nodes and interfering with subsequent immune activation (170, 171). This mechanism may have a dual role in the modulation of psoriasis: it can suppress the spread of inflammation while potentially promoting immune evasion, contributing to the long-term persistence of the condition. NGF has been shown to increase the density of nerve innervation in the epidermis, and changes in this nerve density may affect the distribution and functional state of LCs (172). Research has indicated that the concentration of LCs in the epidermis increases following the removal of nerve innervation, suggesting that the nervous system may regulate LCs indirectly through alterations in the microenvironment.

As antigen-presenting cells in the epidermis, LCs in psoriatic lesions express neuronal markers and neuropeptide receptors (173). They can regulate the sensitivity and density of nerve endings by secreting signaling molecules such as nerve growth factor (174, 175). This interaction may lead to a heightened sensitivity of nerve endings, potentially linking this state to the recurrent flare-ups of psoriasis, making symptoms more challenging to alleviate.

Additionally, peripheral nociceptive neurons associated with LCs secrete SP, which exhibits inhibitory neuroimmune crosstalk (176). This mode of regulation may provide new perspectives and new therapeutic value for understanding the interaction between the nervous system and Langerhans cells in psoriasis.

## Central neuroimmune dysregulation in psoriasis

### Clinical and neuropsychiatric clues linking psoriasis to the CNS

Psoriasis has traditionally been viewed as a peripheral inflammatory skin disease. However, growing evidence links it to central neuroimmune dysfunction. In addition to cutaneous neurogenic inflammation, patients with psoriasis frequently exhibit psychiatric and cognitive manifestations, including depression, anxiety, sleep disturbance (177), and cognitive impairment (178), suggesting that dysregulation of the skin–brain axis may contribute to these comorbidities. This association is unlikely to be explained solely by psychosocial stress secondary to skin lesions. Shared inflammatory and neuroendocrine pathways may also underlie this link.

### Blood–brain barrier disruption and central neuroinflammation

Peripheral inflammation may influence the CNS through cytokine-mediated signaling and blood–brain barrier (BBB) dysfunction. The Th17/IL-17 axis is particularly relevant. IL-17A can disrupt BBB integrity by affecting endothelial barrier function and tight junction pathways and psoriasis-related cytokines such as TNF-α, IL-1β, and IL-6 may further promote endothelial activation (179–182). Once BBB integrity is weakened, peripheral inflammatory signals may activate endothelial cells, astrocytes, microglia, and perivascular immune cells, leading to the production of secondary mediators, including IL-1β, IL-6, TNF-α, and CXCL1, thereby further amplifying local inflammation (183, 184). In this context, psoriasis-related peripheral inflammation may lead to central functional dysregulation, which may help explain cognitive and psychiatric symptoms reported in some patients (178, 185, 186). However, direct evidence for each step of this cascade remains limited, and further studies are needed to clarify how psoriasis-specific cytokine networks alter BBB integrity and induce central immune activation.

Evidence from psoriasis models supports this possibility. IMQ-induced mice exhibit depressive and anxiety behaviors accompanied by altered neurotransmitter levels, microglial activation, and impaired hippocampal neurogenesis (187, 188). In addition, BBB disruption and increased hippocampal IL-17A levels have been observed in psoriatic mice, where IL-17A derived from γδT cells appears to interact with microglia and further amplify hippocampal inflammation (189). Together, these findings link peripheral inflammation in psoriasis to hippocampal injury.

### Central stress circuitry and HPA-axis dysregulation

Stress-related central circuits may provide another link between CNS dysfunction and psoriasis. In psoriasiform mice, altered functional connectivity involving the amygdala has been observed together with anxiety and social avoidance behaviors (190). Clinical evidence is consistent with this view, as improvement in skin disease has been accompanied by reduced amygdala activity (191).

The hypothalamic paraventricular nucleus (PVN) may link stress processing to downstream autonomic and neuroendocrine responses. Recent evidence indicates that the PVN can orchestrate cutaneous inflammation through a brain–sympathetic–skin circuit, highlighting that central circuits may actively shape skin inflammation (192). In psoriasiform mice, inflammation-associated PVN neuronal ensembles appear to encode a form of inflammatory memory, and their reactivation can sustain chronic skin inflammation. Mechanistically, synaptic plasticity within PVN neurons, partly mediated by Eph/ephrin signaling, promotes sympathetic hyperactivity, norepinephrine release, and downstream Th17/Treg imbalance, whereas inhibition of this pathway attenuates these changes and restores skin immune homeostasis (192). Indirect human evidence also suggests that PVN-related pathways may influence stress-endocrine responses. Transcutaneous auricular vagus nerve stimulation may reduce stress-induced cortisol release, and prolonged stimulation is associated with lower diurnal salivary cortisol output (193).

HPA-axis abnormalities further support this brain–skin interaction (194, 195). Altered ACTH/cortisol ratios, abnormal circadian cortisol profiles, and associations between cortisol dynamics, perceived stress, and disease severity have been reported (196, 197). In parallel, a cutaneous HPA-like system composed of CRH family peptides, their receptors, and glucocorticoid-related signaling components is also altered in psoriasis. However, available findings are not fully consistent. CRH-R1 expression has been reported to increase in mast cells and correlate with PASI scores, while other studies suggest downregulation of CRH/CRH-R1 signaling and potential anti-inflammatory effects of CRH (198–200). Genetic studies have also suggested that polymorphisms in CRH-POMC system genes may contribute to psoriasis pathogenesis (201). In addition, IL-17A inhibition has been associated with changes in cortisol levels (202). Together, these findings suggest that stress-related central circuits may interact with systemic and local HPA-axis dysfunction to sustain inflammation in psoriasis.

### Potential of neural interventions

Existing treatments for psoriasis have several limitations. Long-term use of oral medications, such as immunosuppressants, can lead to serious side effects, including hepatic and renal dysfunction, as well as immunosuppression (2, 4, 203, 204). Although biologics more precisely target immune cells or molecules (205–207), they are typically reserved for patients with moderate to severe disease and face challenges such as recurrence after treatment discontinuation (10, 208, 209). Moreover, the effectiveness of drug therapy varies among individuals, with some patients being unresponsive to drugs or developing resistance.

Given the role of the nervous system in regulating inflammation and immune responses in psoriasis, neurointervention has emerged as a promising research area for therapeutic applications. The potential of neurological interventions is increasingly being recognized (**Table 1**), and this approach, which aims to modulate neuro-immune interactions, is expected to play a key role in the treatment of inflammatory diseases such as psoriasis.

**Table 1:** Representative clinical trials related to the nervous system of dermatological diseases.

Target	Potential targeted interactions	Drug	Diseases	Phase	Efficacy
CGRP	Sensory Neuron	Erenumab	Rosacea	Off-label Use Clinical Trials, OLUCTs	The mean (SD) number of days with moderate to extreme flushing was reduced by -6.9 days (95% CI, -10.4 to -3.4 days; P <.001) from 23.6 (5.8) days at baseline. The mean (SD) number of days with moderate to severe erythema was reduced by - 8.1 days (95% CI, -12.5 to - 3.7 days; P <.001) from 15.2 (9.1) days at baseline (210).
		Lidocaine	Psoriatic	Off-label Use of Drugs	Lidocaine treatment markedly reduced patients’ clinical scores (28).
neurokinin 1	SP	Serlopitant	Psoriatic Pruritus	II	The WI-NRS 4-point response rate at 8 weeks (primary end point) was 33.3% for serlopitant vs 21.1% for placebo (P = .028) (211).
TrkA	NGF	CT327	Psoriatic Pruritus	IIb	No effect was found on psoriasis severity using Investigator’s Global Assessment (primary endpoint). clinically and statistically significant reductions in pruritus were observed in the 108 patient subset reporting at least moderate pruritus at baseline (212).
VEGF	Nerve Nutrition and Repair	Neovastat (AE-941)	Plaque Psoriasis	I/II	Improvement in the Psoriasis Area and Severity Index (PASI) score was observed in 50%, 41.7%, and 30.8% of the patients receiving 240, 120, and 60 mL/d, respectively (213).
cAMP	Neuroprotective and anti-inflammatory	Apremilast	Psoriasis Co-morbidity	IV	The drug may have an overall benefit for patients with cardiometabolic disease and psoriasis (214).

Looking at future psoriasis therapies from the perspective of modulating nerves, we believe that sensory neurons can be targeted and modulated by acid-sensing ion channels(ASIC3)-regulated CGRP, which directly affects the proliferation and differentiation of skin keratinocytes and exacerbates local inflammatory responses (63). ASIC3 inhibitors are being looked at in allergic rhinitis (215). In addition, ion channels such as TRPV1 and P2X3 also sense external stimuli and regulate the release of neuropeptides such as CGRP and activate sensory neurons, which then interact with immune cells such as dendritic cells, T cells, and B cells (28, 176), and clinical studies have reported that the TRPV1 antagonist, ACD440 Gel, affects pain in the skin (216). Therefore, we speculate that that specific inhibitors of ASIC3 or related ion channels by topical or systemic administration could also reduce psoriasis symptoms. Blockade of the calcium/calmodulin dependent protein kinase II, gamma(CAMK2γ) pathway reduces sympathetic nerve activity, and potent small-molecule inhibitors targeting CAMK2γ, such as alcaftadine, were shown to be safe and effective in preventing allergic conjunctivitis in a phase 3 clinical trial, and we speculate that they also play a role in the immune response in psoriasis (217).

In addition to directly targeting nerves, treatment of psoriasis can be achieved by targeting key nodes of interaction between nerves and different cells. For example, interactions between sensory neurons and fibroblasts play an important role in disease progression, TrkA promotes fibroblast survival, and among TrkA kinase inhibitors CT327 has been noted to have a role in Psoriatic Pruritus (212), Entrectinib is approved for use with lung cancer (218), and we also look forward to further studies on TrkA inhibitors.

While sensory nerves and DCs act through cAMP, Apremilast, a novel phosphodiesterase 4 (PDE4) inhibitor that regulates inflammation through multiple cAMP downstream effectors, is currently being used to treat psoriasis (219). As clinical trials progress, these new drugs or targets show great potential to play a role in the treatment of psoriasis.

### TCM-derived interventions targeting neuroimmune-related pathways

Traditional Chinese medicine (TCM)-derived compounds have also been investigated as modulators of inflammatory and neuroimmune pathways relevant to psoriasis (**Table 2**). Terrestrosin D, an active component of *Tribulus terrestris* L., reduced SP expression, DC maturation, and IL-12p70 and IL-23 production, while improving skin lesions and behavioral abnormalities in IMQ-induced psoriasiform mice (220). At the level of sensory ion channels, sophocarpine from *Sophorae Flavescentis* Radix reduced TRPA1/TRPV1 expression in the trigeminal ganglion and attenuated inflammatory itch and pain in a murine allergic contact dermatitis model (221). Honokiol and magnolol, major bioactive constituents of *Magnolia officinalis*, inhibited TRPV3-mediated Ca²^+^ influx and IL-6/IL-8 release in human keratinocytes (222). Zinc-doped carbon dots derived from *Isatis indigotica* Folium and calamine also ameliorated IMQ-induced psoriasiform dermatitis by reducing oxidative stress and intracellular Ca²^+^ levels and suppressing NLRP3 inflammasome activation, pyroptosis, and MAPK/NF-κB signaling (223).

**Table 2:** Representative TCM-derived compounds targeting neuroimmune-related pathways.

TCM-derived compound	Source of origin	Potential targets
Terrestrosin D (220)	*Tribulus terrestris* L.	SP; DC maturation; IL-12p70; IL-23
Sophocarpine (221)	*Sophorae Flavescentis Radix*	TRPA1; TRPV1
Honokiol (222)	*Magnolia officinalis*	TRPV3; Ca²^+^ influx; IL-6/IL-8
Magnolol (222)	*Magnolia officinalis*	TRPV3; Ca²^+^ influx; IL-6/IL-8
Zinc-doped carbon dots (223)	*Isatis indigotica* Folium and calamine	Intracellular Ca²^+^; NLRP3 inflammasome; MAPK/NF-κB signaling

Together, these findings suggest that TCM-derived compounds may modulate neuropeptide signaling, sensory TRP channels, and Ca²^+^-dependent inflammatory pathways relevant to psoriasis. However, studies directly examining their effects on neuroimmune interactions in psoriasis remain limited, and current evidence is predominantly preclinical.

Advances in our understanding of neural regulation in cutaneous inflammation have opened new avenues for intervention, ranging from targeting neuropeptides and ion channels to modulating specific nerve–cell interaction pathways. These approaches may not only improve disease control but also address symptoms such as pruritus and pain that are inadequately managed by conventional therapies. In this context, several critical research questions arise:

1. How neural signals differentially regulate distinct immune cell subsets, particularly Th17 cells and DCs, in the psoriatic microenvironment remains to be elucidated.
2. The extent to which alterations in peripheral versus central nervous system pathways contribute to psoriasis initiation, progression, and relapse warrants further investigation.
3. Whether targeting neuro-immune interaction pathways can provide sustained therapeutic benefits compared with current immune-focused biologics remains an open question.
4. How non-immune skin cells, such as keratinocytes and fibroblasts, integrate neural signals to amplify or resolve local inflammation is not yet fully understood.

## Summary and prospects

In future research on psoriasis and other autoimmune diseases, further exploration of the neuro-immune interaction mechanisms will be of great significance. The network regulation of neuro-immune interactions and the application of neuromodulation in the treatment of autoimmune diseases remain worth exploring. This will also drive interdisciplinary collaboration, enabling a more comprehensive and profound understanding of the pathogenesis and treatment of psoriasis.

In conclusion, the mechanism of neural involvement in psoriasis and other autoimmune diseases represents a complex yet vital research area. Future studies will further unravel the mysteries of this field and bring better treatment options and improved quality of life for patients.
